# Maternal Postnatal Depression and Anxiety and Their Association With Child Emotional Negativity and Behavior Problems at Two Years

**DOI:** 10.1037/dev0000221

**Published:** 2017-01

**Authors:** Jason M. Prenoveau, Michelle G. Craske, Valerie West, Andreas Giannakakis, Maria Zioga, Annukka Lehtonen, Beverley Davies, Elena Netsi, Jessica Cardy, Peter Cooper, Lynne Murray, Alan Stein

**Affiliations:** 1 Department of Psychology, Loyola University Maryland; 2 Department of Psychology and Department of Psychiatry and Biobehavioral Sciences, University of California, Los Angeles; 3 Department of Psychiatry, University of Oxford; 4 Department of Psychology, University of Reading, and Department of Psychology, Stellenbosch University; 5 Department of Psychology, University of Reading, and Department of Psychology, University of Cape Town; 6 Department of Psychiatry, University of Oxford

**Keywords:** maternal depression, maternal generalized anxiety disorder, postnatal, emotional negativity

## Abstract

Postnatal maternal depression is associated with poorer child emotional and behavioral functioning, but it is unclear whether this occurs following brief episodes or only with persistent depression. Little research has examined the relation between postnatal anxiety and child outcomes. The present study examined the role of postnatal major depressive disorder (MDD) and generalized anxiety disorder (GAD) symptom chronicity on children’s emotional and behavioral functioning at 24 months. Following postnatal screening mothers (*n* = 296) were identified as having MDD, GAD, MDD and GAD, or no disorder at 3 months postnatal; the average age was 32.3 (*SD* = 5.0), 91.9% self-identified as Caucasian, and 62.2% were married. Maternal disorder symptom severity was assessed by questionnaires and structured interview at 3, 6, 10, 14, and 24 months postpartum. At 24 months, child emotional negativity and behavior were assessed using questionnaires and by direct observation. Latent trait–state-occasion modeling was used to represent maternal disorder symptom chronicity; both stable trait and time-specific occasion portions of maternal symptomatology were examined in relation to child outcomes. Only the stable trait portion of maternal MDD and GAD symptom severity were related to maternal report of child behavior problems and higher levels of emotional negativity. Persistent maternal MDD, but not GAD, symptom severity was related to higher levels of child emotional negativity as measured observationally. These data suggest that children’s behavior problems and emotional negativity are adversely affected by persistent maternal depression, and possibly anxiety. This has implications for interventions to prevent negative effects of postnatal psychopathology on children.

Parental psychopathology in a child’s early years is associated with adverse effects on infant and child development. The parental disorder that has received the most attention in this regard is postnatal depression, which affects up to 19% of mothers in developed countries (Gavin et al., 2005). A plethora of evidence demonstrates the adverse effects of maternal depression on children’s cognitive, emotional, and behavioral development (for a meta-analytic review, see Goodman et al., 2011; for a review, see Murray, Halligan, & Cooper, 2010; Stein et al., 2014). Although the majority of early studies were cross-sectional in nature, a growing body of literature has shown longitudinal associations between maternal depression and child development (e.g., Brennan et al., 2000; Carter, Garrity-Rokous, Chazan-Cohen, Little, & Briggs-Gowan, 2001; Cornish et al., 2005; National Institute of Child Health and Human Development [NICHD] Early Child Care Research Network, 1999).

A key factor believed to moderate the effect of maternal depression on child outcomes is chronicity of psychopathology. Persistence of maternal disorders predicts later problems in child development, whereas brief postnatal depressive episodes have less impact (e.g., Brennan et al., 2000; Cornish et al., 2005; NICHD Early Child Care Research Network, 1999). Specifically, when maternal depression was assessed at 1, 6, 15, 24, and 36 months postpartum and child outcomes were assessed at 36 months postpartum, child outcomes were poorest for those mothers classified as chronically depressed (deemed depressed at four or five assessments) compared with those either sometimes depressed (deemed depressed at less than four assessments) or never depressed (NICHD Early Child Care Research Network, 1999). Whereas maternal depression at 2 months postpartum was not associated with impaired mother-infant interaction or infant response during face-to-face interaction, mothers with chronic depression through 6 months postpartum were less positive with their infants during face-to-face interactions and their infants were in turn less positive with them (Campbell, Cohn, & Meyers, 1995). Such lower levels of maternal positive affect expression and contingent maternal responsiveness are hypothesized to play a role in child development of affect regulation and dyadic communication (Tronick, 1989).

Another study found that child developmental outcomes at 15 months postpartum were poorest for children of mothers whose depression lasted throughout the first 12 postpartum months and beyond; there were no differences in child outcomes when comparing mothers who were never depressed and those whose depression did not last through 12 months (Cornish et al., 2005). However, mothers with chronic depression also had more severe depression at all time points compared with those with nonchronic depression.

Brennan and colleagues (2000) attempted to disentangle the contributions of maternal depressive severity and chronicity by simultaneously examining continuous measures of both constructs, and categorizing mothers into four groups: neither severe nor chronically depressed, chronically but not severely depressed, severely but not chronically depressed, and both severely and chronically depressed. They observed a significant interaction between chronicity and severity in the prediction of child problems (as assessed by the total problems scale on the Child Behavior Checklist). Only those children with both severely and chronically depressed mothers had significantly higher problems compared with the other groups (Brennan et al., 2000).

Another potential moderator of maternal depression on child outcomes is timing of maternal depression. There are likely to be certain periods during infant and child development when exposure to experiences associated with maternal depression have more, or less, impact on brain and behavioral development (e.g., Dawson, Ashman, & Carver, 2000). One of the few studies to longitudinally examine the relation between timing of maternal depression and child outcomes examined mothers who had elevated depression at a single assessment only in order to avoid confounding timing with severity and chronicity (Brennan et al., 2000). Findings revealed that child outcomes were significantly poorer for mothers who were depressed only at 6 months postpartum compared with those who were only depressed during pregnancy or only depressed shortly after birth of their child.

The role that anxiety disorders play with respect to child developmental outcomes is another important consideration, given that anxiety disorders occur in 4%-8% of mothers in the postnatal period (Ross & McLean, 2006), and often co-occur with depression. Although less studied than maternal depression, maternal anxiety alone or in combination with depression may be associated with difficulties in mother–child interactions and negatively affect child outcomes (Nicol-Harper, Harvey, & Stein, 2007; Murray, Cooper, Creswell, Schofield, & Sack, 2007, 2008). Longitudinal research on anxiety and child developmental outcomes is more limited, but at least one study has found that poorer child outcomes are associated with more persistent maternal anxiety (Spence, Najman, Bor, O’Callaghan, & Williams, 2002). Further, despite the high comorbidity of depression and anxiety, and the fact that comorbidity of psychopathology is associated with poorer functioning in adults (e.g., Kessler et al., 1996; Mineka et al., 1998), there has been little investigation of the impact of such comorbidity and child outcomes. At least one study has demonstrated that the relation between maternal depression and poorer mother—infant interactions and insecure infant attachment was specific to those mothers with comorbid diagnoses (Carter et al., 2001).

There are a number of limitations with the existing literature examining the relation between chronicity of maternal psychopathology and child outcomes. There are few studies that attempt to disentangle chronicity of maternal psychopathology from severity (e.g., Brennan et al., 2000) and as noted above, there has also been little investigation of the relation between maternal anxiety and child outcomes. Many of these studies use symptom measure cut-off scores to categorize mothers as depressed or nondepressed (e.g., Brennan et al., 2000; NICHD Early Child Care Research Network, 1999). This method of assessing depression is a limitation of prior studies because it does not account for the fact that scores on manifest variables contain random measurement error. Another limitation of this method is that although questionnaires provide continuous data, the approaches above dichotomize this data which will not accurately reflect psychopathological variables if they are dimensional in nature (Brown, Chorpita, & Barlow, 1998). Perhaps the more important limitation of existing literature is that chronicity has typically been operationalized in a fairly simplistic manner; for example, by dividing mothers into groups based on the number of time points they were depressed (e.g., never depressed, sometimes depressed, and chronically depressed; NICHD Early Child Care Research Network, 1999), or by creating a continuous variable to represent the number of times the mother was depressed (e.g., Brennan et al., 2000). Such approaches to operationalizing chronicity may not reflect the manner in which psychopathological temporal stability is expressed (e.g., Cole, 2006).

Many psychopathological constructs, including symptoms of depression (e.g., Holsen, Kraft, & Vitterso, 2000) and anxiety (Gullone, King, & Ollendick, 2001), display decreases in relative stability with increasing time elapsed between measurement points, but their relative stability does not decrease to zero even over long intervals between measurement points. The latent trait–state–occasion (TSO; Cole, Martin, & Steiger, 2005) model is ideal for representing this pattern of relative stability expression (see Figure 1). The TSO model contains a time-invariant trait factor that allows state factors to be correlated at all time points, accounting for the observation that relative stability does not decrease to zero even over long intervals between measurement points. If relative stability increases with decreasing time elapsed between measurement points, state factors at different time points will be related even after accounting for the trait factor. Adjacent time points will be the most strongly related and the relation will decrease as the time points under consideration become further apart. The TSO model accounts for this with autoregressive pathways between occasion factors (see Figure 1) that allow relative standing on the construct at different time points to be related above and beyond that accounted for by the trait factor; these pathways model increasing stability with decreasing intervals between measurement points.[Fig-anchor fig1]

TSO modeling enables decomposition of standing on the construct under consideration, depression and anxiety symptoms in this case, at each time point (the state factors, see Figure 1) into a component that is free of situational (the trait factor) and a situational component (the occasion factor). Thus, psychopathological chronicity for the time period under consideration is represented by the trait factor. An individual’s score on the continuous trait factor indicates the extent to which they are chronically at the highest standing on depression (or anxiety) relative to others, chronically at the lowest standing on depression (or anxiety) relative to others, or chronically anywhere between. Individual’s scores on the continuous occasion factors represents time-point-specific fluctuations above or below their trait standing. Unlike most other methods used in past studies of chronicity of maternal psychopathology, the TSO model does not confound chronicity with severity. Both the chronic, trait factor and the more transient, occasion factors provide continuous representations of maternal symptom severity.

Thus, the TSO model can be used to improve efforts at identifying predictor variables in risk-outcome longitudinal studies (Cole & Maxwell, 2009). Specifically, because the TSO model can separate the time-varying and time-invariant portions of the predictor construct (as noted above), the outcome under consideration can be predicted separately from the time-varying and time-invariant components of the predictor (e.g., LaGrange et al., 2011). Separation of the time-varying and time-invariant components results in more meaningful statistical tests and effect sizes (Cole, 2006). Thus, for that portion of depressive (or anxiety) symptoms that are time-invariant, or stable with time, (represented by the trait factor), the TSO model permits examination of the relation between stable, chronic symptom severity and child outcomes. Likewise, for that portion of depressive (or anxiety) symptoms that are transient with time, time-varying, (represented by the occasion factor), the TSO model permits examination of the relation between transient symptom severity at each time point and child outcomes. For additional information about latent TSO models and their interpretation, see Prenoveau (2016).

The present study’s aim was to examine whether maternal postnatal depression and anxiety symptom severity are associated with children’s emotional negativity and behavior at 24 months. In doing so, the present study sought to address a number of limitations with the existing literature examining the relation between chronicity of maternal symptomatology and child outcomes. Specifically, maternal anxiety was examined (in addition to maternal depression) because little research has examined maternal anxiety in this context despite the high rates of anxiety during the postnatal period and the fact that anxiety may be associated with difficulties in mother–child interactions. Also, latent variable TSO modeling (see Figure 1) was employed to represent the chronicity of depression and anxiety with time. Such modeling enables an integrative approach to addressing a number of limitations with past research in this area.

First, the use of latent constructs in the TSO models accounts for measurement error as well as measure-specific effects over time, which can yield more accurate parameter estimates. Second, both self-report questionnaires and semistructured interviews were used as indicators of TSO latent constructs to address the limitation of past studies that dichotomization will not accurately reflect psychopathological variables that are dimensional in nature (Brown et al., 1998). Third, in contrast to methods used for operationalizing chronicity in past studies, the TSO model is ideal for representing the pattern of relative stability expression observed for symptoms of both depression and anxiety, resulting in more meaningful statistical tests and effect sizes (Cole, 2006).

TSO modeling was used to examine whether or not the chronic component of maternal symptomatology, the trait factor, would predict child outcomes above and beyond that predicted by the occasion specific levels of maternal symptomatology. In other words, is the coefficient for a child outcome regressed on the trait factor (β_*T*_, Figure 2) significant when also regressing the child outcome on the occasion factors. Additionally, to determine if there are sensitive periods during development regarding the child effects of exposure to maternal symptomatology, maternal symptomatology was examined at a number of occasions: 3, 6, 10, 14, and 24 months postpartum) within the TSO framework. In other words, is the coefficient for a child outcome regressed on an occasion factor at a specific time point (e.g., β_*O*_3*M*-*P*__, Figure 2) significant when also regressing the child outcome on the trait factor.[Fig-anchor fig2]

Mothers were assessed at 3 months postpartum as many episodes of depression have their onset within this period. A significant proportion remit by 6 months postpartum, so this was chosen for the second maternal assessment to distinguish those with brief elevations in maternal symptomatology compared with those that may become more chronic. The 10-month postpartum assessment was chosen based on the needs of the larger Oxford Parent Project because it is a time when child language begins to develop as well as social interaction between mother and infant. At 14 months postpartum, the child has begun to walk, exploratory behavior increases, and attachment behavior is at its peak; this is also a time point where it can be ascertained whether or not elevated maternal symptomatology has persisted beyond the postnatal period.

Child outcomes were assessed at 24 months because by this age children are more independent and are able to deploy self-regulating strategies and to focus their attention away from stressful stimuli in order to manage their distress as well as to soothe themselves (Spinrad, Stifter, Donelan-McCall, & Turner, 2004). At the same time by 24 months of age children still generally need support in dealing with frustrating situations in order to regulate their own emotions (Dennis, 2006). At this stage children also have sufficient language skills to communicate with others which helps with managing their negative emotions (Thomson & Meyer, 2007). Additionally, higher levels of negative emotional reactivity as early as 21 months is related to poorer emotional development later in childhood (e.g., Kagan, Snidman, & Arcus, 1998).

Consistent with past research (e.g., Brennan et al., 2000; NICHD Early Child Care Research Network, 1999), a total problems score on the Child Behavior Checklist was used as a measure of child outcomes in order to capture a broad range of emotional and behavioral problems. In addition to child depression and other internalizing problems, maternal depression has also been shown to be associated with elevated rates of externalizing problems in offspring (Goodman et al., 2011). Thus, both internalizing and externalizing problems were examined as child outcomes in the present study to compare the relative risk for these problems for maternal depressive and anxious symptomatology. As noted above, lower levels of maternal positive affect expression and contingent maternal responsiveness associated with maternal depression are hypothesized to play a role in child development of affect regulation and dyadic communication (Tronick, 1989). Thus, child outcomes related to the development of affect regulation were also assessed, including: attention, frustration, soothability, attentional focusing, attentional shifting, negative emotional reactivity, and negative emotional tone.

Maternal symptom severity for depression and anxiety were examined separately as well as together to determine if one predicted child outcomes after accounting for the other. Based on prior work (e.g., Brennan et al., 2000; Cornish et al., 2005; NICHD Early Child Care Research Network, 1999; Nicol-Harper et al., 2007; Murray et al., 2007, 2008), it was predicted that depression and anxiety in the postnatal period would predict poorer child emotional negativity and behavioral outcomes and that only the chronic component of maternal symptomatology would be related to child outcomes. Thus, it was hypothesized that maternal depression and anxiety trait factors (the stable, chronic component of maternal symptomatology), but not occasion factors, would be related to child outcomes, such that higher trait factor scores would be associated with poorer child motional negativity and poorer behavioral outcomes.

## Method

### Participants

As part of the larger Oxford Parent Project, mothers were recruited from an Oxford Hospital’s postnatal wards and health centers in Oxfordshire. To be eligible, mothers had to be 18 years or older, speak sufficient English to participate, live within 35 miles of Oxford, have no life-threatening medical conditions, and plan to be the primary caregiver. Infants had to be delivered over 35 weeks gestation, weighing over 2,000 g, and with no life-threatening medical complications. Study approval was obtained from the Oxford Research Ethics Committee. Informed consent was obtained from all mothers for themselves and their child.

Two-thousand two-hundred and two mothers completed the Edinburgh Postnatal Depression Scale (EPDS; Cox, Holden, & Sagovsky, 1987) and Generalized Anxiety Disorder Questionnaire (GAD-Q; Newman et al., 2002) approximately 9 weeks postpartum to identify those likely to have major depressive disorder (MDD), GAD, or both. To ensure adequate numbers of participants with, and without, GAD and MDD for the larger Oxford Parent Project, mothers scoring above either screening questionnaire cut-off (>12 on the EPDS, >5.70 on the GAD-Q), and a randomly selected group of women who scored below cut-offs, were selected for diagnostic interviews at 3 months postpartum. Those who scored above the cut-offs included 472 who scored above the GAD-Q cut-off and 293 who scored above the EPDS cut-off (226 of these scored above both cut-offs). From those who scored about the cut-offs, we selected all those who fulfilled a *Diagnostic and Statistical Manual of Mental Disorders* (4th ed.; *DSM–IV*; American Psychiatric Association, 2000) diagnosis for MDD, GAD, or both MDD and GAD. From those who scored below the cut-offs, we selected those who the interviews confirmed did not meet criteria for either disorder.

Of the 296 participants who were included in the final sample following diagnostic interviews at 3 months postpartum, 272 (91.9%) self-identified as Caucasian, 269 (90.9%) reported that English was the main language with their baby, 184 (62.2%) reported being married, and 180 (60.8%) indicated that this was their first child. The average mother’s age at the 3-month postpartum assessment was 32.3 years (*SD* = 5.0) and the average child age was 3.6 months (*SD* = 0.8). Although demographic information is presented separately by diagnostic status at 3 months postpartum in Table 1 for informational purposes, predictor variables for analyses described below are continuous measures of MDD and GAD symptom severity as opposed to dichotomous variables representing solely presence or absence of disorder.[Table-anchor tbl1]

### Procedure

At 9 weeks postpartum, the EPDS and GAD-Q were administered to mothers to screen for symptoms of MDD and GAD. At 3 months postpartum the Structured Clinical Interview for *DSM–IV* Disorders was administered to mothers in their homes to assess the presence or absence of MDD and GAD as well as associated Clinician Severity Ratings (described below in Measures). At subsequent assessments (6, 10, 14, and 24 months postpartum), mothers completed the EPDS and GAD-Q and were reinterviewed using the Structured Clinical Interview for *DSM–IV* Disorders. The following number of mothers provided data: 296 at 6 months postpartum, 253 at 10 months postpartum, 233 at14 months postpartum, and 234 at 24 months postpartum.

At 24 months postpartum, in addition to the maternal assessment described above, mothers also completed the Child Behavior Checklist for Ages 1.5–5 and Early Childhood Behavior Questionnaire (described below in Measures). Also at 24 months postpartum, mothers and children participated in a standardized semistructured mother–child interaction play task for 10 min (e.g., NICHD Early Child Care Research Network, 1999). The mother–child interaction play task involved mother and child playing together with a toy farm set. The farm roof and door were given to the mother and child to assemble as they wished, and several rubber farm animals, a plastic tractor, four sets of wooden fences, and a wooden ladder were also provided. Upon completion of the mother–child interaction play task, the child completed a standardized semistructured individual play task: the child continuing to play with the farm set on his or her own for 5 min. while his or her mother completed questionnaires nearby.

Also at 24 months postpartum, children participated in a frustration inducing toy removal procedure, called the barrier paradigm, that originated from the Laboratory Temperament Assessment Battery (Goldsmith & Rothbart, 1991). Such toy removal procedures have been successfully used to elicit frustration in 24-month-old children (e.g., Blair et al., 2015; Diener & Mangelsdorf, 1999). Mother and child sat side by side at a table with the researcher opposite the mother. The mother was asked not to interact with or respond to the child. The child was given a toy to explore for 15 seconds; the toy was then placed behind a transparent screen located at arm’s length from the child for 30 seconds. The child could see but not reach the toy. The toy was then removed and this process repeated for two additional toys. From the child’s perspective, each play session terminated unexpectedly and the child had to cope with the toy being taken away while still visible.

### Measures

#### Maternal MDD

The Structured Clinical Interview for *DSM–IV* Disorders is a semistructured interview for diagnosing psychiatric disorders, which has demonstrated good reliability and validity (First, Spitzer, Gibbon, & Williams, 2002). Clinical severity ratings provide a measure of the extent of symptom severity, distress and impairment associated with psychiatric disorders, using a 0- to 8-point scale. Ratings of 4 or greater indicate clinical severity, whereas ratings of 2 or 3 indicate subclinical symptom severity. Clinical severity ratings have demonstrated good interrater reliability (Brown, Di Nardo, Lehman, & Campbell, 2001). For the present study, all interviews were conducted by extensively trained interviewers and were audiorecorded. Throughout the study, interviewers attended weekly case meetings (led by Alan Stein) for supervision and review of diagnostic assessments. After each interviewer completed five to six interviews, an independent interviewer corated the next interview to assure interrater reliability. Michelle G. Craske reviewed tapes of randomly selected interviews during the first 6 months of the study and continued to review tapes periodically throughout the study. These steps were taken to increase interrater reliability and minimize interviewer drift.

**Figure 1 fig1:**
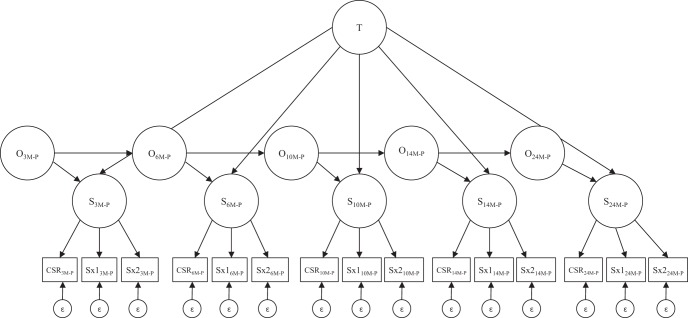
Graphical depiction of the trait–state-occasion model for a construct, either symptoms of generalized anxiety disorder (GAD) or major depressive disorder (MDD), measured by three manifest indicators (CSR, Sx1, Sx2) at five time points (3M-P, 6M-P, 10M-P, 14M-P, 24M-P). T = trait factor; O = occasion factor; S = state factor; CSR = clinician severity rating; Sx1 and Sx2 represent GAD and MDD subscales discussed in Measures; 3M-P = 3 months postpartum; 6M-P = 6 months postpartum; 10M-P = 10 months postpartum; 14M-P = 14 months postpartum; 24M-P = 24 months postpartum.

Interview assessed MDD clinical severity ratings served as one indicator for the latent variable representing maternal MDD symptom severity. The EPDS, a well-validated 10-item questionnaire assessing MDD symptoms in the postnatal period, was divided into two subscales that also served as maternal MDD symptom severity latent variable indicators. Continuous subscales were used as indicators rather than using all 10 categorical items to decrease the number of estimated parameters. Two subscales were created (rather than using the single EPDS total score) to provide each latent construct with three indicators because having three indicators improves model convergence rates (see Prenoveau et al., 2013); these subscales are referred to as Sx_1_ and Sx_2_ in Figure 1 and the Data Analysis section. These subscales had alpha reliability estimates of .80 and .82 at 3 months postpartum.

#### Maternal GAD

Interview assessed GAD clinical severity ratings served as one indicator of the latent variable representing maternal GAD symptom severity. GAD clinical severity ratings were measured using the same 0- to 8-point scale discussed above for maternal MDD, with ratings of 4 or greater indicating clinical severity and ratings of 2 or 3 indicating subclinical symptom severity. The GAD-Q, a well-validated 9-item questionnaire that assesses GAD symptoms, was divided into two subscales that also served as maternal GAD latent variable indicators symptom severity. As above, two subscales were used to decrease the number of parameters estimated and improve model convergence rates; as with maternal MDD, these subscales are referred to as Sx_1_ and Sx_2_ (for GAD symptoms) in Figure 1 and the Data Analysis section. These subscales had alpha reliability estimates of .85 and .88 at 3 months postpartum.

#### Child Behavior Checklist for Ages 1.5–5 (CBCL)

The CBCL (Achenbach, Edelbrock, & Howell, 1987), a well-validated 103-item questionnaire, was completed by the mother. The CBCL assessed children’s emotional and behavioral problems and competencies in the two months prior to the 24 months postpartum assessment. In addition to the CBCL total score (total problems), three subscales were used for the current analyses: attention, internalizing, and externalizing problems. The internalizing subscale measures behaviors related to anxiety and depression, whereas the externalizing subscale measures behaviors related to aggression, hyperactivity, and noncompliance. The attention subscale assesses behaviors related to attention problems such as difficulty maintaining attention.

#### Early Childhood Behavior Questionnaire (ECBQ)

The ECBQ is a well validated instrument for assessing temperament in children between the ages of 18 and 36 months. Items from four dimensions of the ECBQ (Putnam, Gartstein, & Rothbart, 2006) were administered to mothers to assess key components of child temperament: child soothability, frustration, attentional focusing, and attentional shifting. Child soothability refers to the rate of recovery from distress, excitement, or general arousal. Frustration is the negative affect that is related to the interruption of ongoing tasks. Soothablity and frustration are both indicators of negative emotional reactivity. Attentional focusing refers to the ability to sustain orienting on an object of attention, or to resist distraction. Attentional shifting is the ability to transfer focus of attention from one activity to another.

#### Observational measures

Two cameras recorded the mother–child interaction play task, child individual play task, and barrier paradigm. The cameras had different viewing angles connected to a split-screen generator to permit precise coding. Videotaped recordings of each interaction were coded by a single rater blind to maternal diagnostic status and study hypotheses. The rater was trained to reliability with a gold-standard rater prior to rating study videotapes. Checks were made periodically to ensure that there was no drift. A set of behaviors reflecting the constructs being examined were rated on a predefined ordinal scale; scores derived in this manner for the constructs assessed below have demonstrated adequate reliability and validity (e.g., Murray, Fiori-Cowley, Hooper, & Cooper, 1996; Stein et al., 2012; Stein, Woolley, Cooper, & Fairburn, 1994). Cases were randomly selected and coded by a second rater for reliability purposes. Square-weighted Kappa (κ) values (provided below) demonstrated good interrater reliability for all of the observational measures.

##### Child negative emotional reactivity

Child negative emotional reactivity was coded in response to each play termination during the barrier paradigm on a scale ranging from 1 (*very distressed*) to 5 (*calm*); these three measurements were averaged as the index of child negative emotional reactivity. Ratings were based on the extent to which children remained calm or displayed distress in response to the frustrating task (play termination). There is evidence that child responses to such toy removal procedures index negative emotional reactivity (Blair et al., 2015). Interrater reliability for child negative emotional reactivity was κ = .786.

##### Child negative emotional tone

Child negative emotional tone was coded on a scale from 1 (*very unhappy*) to 5 (*very happy*) with ratings made on the basis of vocalizations, facial expressions, and behavioral responses during the first two play tasks and after each play termination during the barrier paradigm. These five measurements were averaged as the index of child negative emotional tone. Such scores have evidenced reliability and validity at assessing child negative emotional tone (e.g., Stein et al., 1994). Interrater reliability for child negative emotional tone was κ = .775.

##### Maternal sensitivity

Maternal sensitivity toward their child was coded on a scale ranging from 1 (*not at all sensitive to child’s needs*) to 5 (*highly sensitive*). Ratings were based on the extent to which the mother responded to her child’s signals and communications, taking account of whether her responses were appropriate, prompt, and warm in manner. Maternal sensitivity was coded once every 5 min. during the first play task and these two measurements were averaged as the index of maternal sensitivity. Such scores have evidenced reliability and validity at assessing maternal sensitivity (e.g., Halligan et al., 2013; Murray et al., 1996). Interrater reliability for maternal sensitivity was κ = .716.

#### Data analysis

Mplus version 5.0 statistical software (Muthén & Muthén, 1998) was used for structural equation modeling. Participant attrition was not significantly predicted from 3 months postpartum GAD or MDD symptom severity. Thus, missing data were accommodated using full information maximum-likelihood under the assumption of missing at random. Model goodness of fit was evaluated using the root mean square error of approximation (RMSEA) and the comparative fit index (CFI). To conclude good fit between the observed data and hypothesized model, RMSEA should be less than .06 and CFI should be greater than .95 (Hu & Bentler, 1998; Yu, 2002).

First, descriptive statistics for all study variables will be examined, including variable means, standard deviations, and correlations among all study variables. Next, longitudinal measurement models will be examined separately for maternal MDD and GAD symptoms to ensure that manifest variables are significant indicators of their latent constructs at each of the five time points. The bottom half of Figure 1 illustrates the state latent variables (S_*3M-P*_ *to S*_*24M-P*_*)* and their corresponding manifest variable indicators *(clinical severity ratings, Sx*_*1*_*, and Sx*_*2*_*)* at each time point. For maternal MDD symptom severity, *S*_*t*_ represents state standing on the MDD symptom severity latent variable at time-point *t*, indicated by MDD clinical severity ratings and the EPDS subscales (described above) at time *t*. Similarly, for maternal GAD symptom severity, S_t_ represents state standing on the GAD symptom severity latent variable at time-point *t*, indicated by GAD clinical severity ratings and GAD-Q subscales (described above) at time *t*. Removal of the top half of Figure 1 (the O and T latent variables), and the addition of pathways allowing correlations among all of the state latent variables at each time-point results in longitudinal measurement models for MDD and GAD symptom severity. If longitudinal measurement models for MDD and GAD symptom severity fit the data well, then metric invariance with time will be examined to determine if latent construct indicators function the same way at each point. This is done to ensure that actual change in the latent construct with time is not confounded by change in the measurement of the construct with time.

Next, full TSO models (see Figure 1) will be examined by removing correlational pathways among the state latent variables from the longitudinal measurement models and including autoregressive occasion factors at each time point (O_t_) and a stable trait factor (T). TSO model fit will be examined separately for maternal MDD and GAD symptoms. If TSO models for MDD and GAD symptom severity fit the data well, information on the chronicity of maternal MDD and GAD symptoms will be provided. Specifically, the TSO model enables variance in state standing (S_t_) of symptom severity at each time point to be completely partitioned into that explained by standing on the stable trait factor (symptom chronicity), and that which is not chronic throughout the period under consideration: that explained through the prior occasion factor through the autoregressive pathway and that unexplained by knowing state standing at other time points.

Next, for descriptive purposes, individuals will be grouped based on their continuous MDD and GAD symptom severity trait factor scores. Specifically, four groups will be created for each disorder by dividing individuals into quartiles based on their trait factor scores. Then, for each of these groups, information will be provided about average clinical severity ratings across all time points and number of time points with MDD and GAD. This information is purely descriptive in nature and is meant to provide insight into how the TSO model represents chronicity by providing descriptive information about those who score at different levels on factor representing chronicity in the model, the trait factor.

**Figure 2 fig2:**
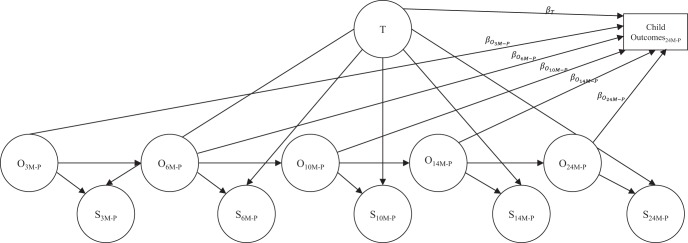
Child outcomes at 24 months postpartum predicted by maternal symptomatology as represented by the trait–state–occasion model. Symptoms of generalized anxiety disorder (GAD) and major depressive disorder (MDD) were modeled separately; state factor manifest variable indicators were excluded for clarity (these are included in Figure 1). Although not depicted for clarity purposes, marital status and infant birth order were also included as predictors of child outcomes because they were potentially confounded with MDD and GAD symptoms severity. T = trait factor; O = occasion factor; S = state factor; 3M-P = 3 months postpartum; 6M-P = 6 months postpartum; 10M-P = 10 months postpartum; 14M-P = 14 months postpartum; 24M-P = 24 months postpartum.

Key study hypotheses will be tested next using regression analyses conducted within structural equation modeling and using the TSO framework. Specifically, each child outcome will be examined independently of the other outcomes and separate models will be tested for maternal MDD and GAD symptom severity; all models will first be examined to determine if they fit the data well. To examine the hypothesis that that the chronic component of maternal symptomatology would be related to child outcomes, each child outcome at 24 months postpartum will be regressed onto the trait factor (β_*T*_ in Figure 2). This will be done while simultaneously regressing each child outcome at 24 months postpartum on all of the occasion factors (β_*O*_3*M*-*P*__, β_*O*_6*M*-*P*__, β_*O*_10*M*-*P*__, β_*O*_14*M*-*P*__, β_*O*_24*M*-*P*__, Figure 2) to examine whether or not there are sensitive periods during development regarding child effects of exposure to maternal symptomatology (it is not hypothesized that there will be). Thus, each Figure 2 regression pathway represents the unique relation between a TSO model component and child outcome, when accounting for the relations between the other TSO model components and the child outcome.

As seen in Table 1, marital status and infant birth order differed significantly between diagnostic status groups. Given that these variables are potentially confounded with measures of MDD and GAD symptom severity, they will be included as predictors of child outcomes in all models. However, they have not been included in Figure 2 for clarity purposes.

For child outcomes that are found to be significantly predicted by both MDD and GAD TSO components, MDD and GAD TSO models will be combined in a single model to determine if MDD symptom severity independently predicts these child outcomes when accounting for GAD symptom severity, and if GAD symptom severity independently predicts these child outcomes when accounting for MDD symptom severity. Given the known strong relation between MDD and GAD, trait factors will be allowed to correlate with one another, as will MDD and GAD occasion factors at each time point. If this model is a good fit to the data, the relevant child outcomes at 24 months postpartum will be simultaneously regressed onto the MDD and GAD TSO components that were significantly predictive of the child outcome when examined separately.

### Results

Correlations among maternal symptom severity manifest variables at 3, 6, 10, 14, and 24 months postpartum and child outcomes at 24 months postpartum, as well as means and standard deviations for these variables, can be accessed as supplementary material (a legible table including this data could not be included herein because of the large number of variables involved). Longitudinal measurement models fit the data well for both maternal symptoms of MDD, CFI = .99, RMSEA = .045, and GAD, CFI = .99, RMSEA = .038. Both measurement models displayed partial metric invariance with time, indicating that change in the latent construct with time is not confounded by change in measurement of the construct with time.

TSO models fit the data well for both maternal MDD, CFI = .98, RMSEA = .043, and GAD, CFI = .99, RMSEA = .036. About 54% of the variance in maternal MDD symptom severity state standing (S_t_) is explained by standing on the stable trait factor, whereas 9.5% is explained by the prior occasion factor through the autoregressive pathway, and 36.4% is unexplained. Maternal GAD symptom severity is more stable, or chronic, during the period under consideration with 76.5% of the state variance explained by standing on the stable trait factor, 2.0% through the autoregressive pathway, and 21.5% is unexplained.

Table 2 provides information about average clinical severity ratings across all time points and number of time points with MDD and GAD for groups of mothers based on their standing on the trait factors of MDD and GAD symptom severity. As seen in Table 2, the average number of time points with a diagnosis of GAD increases from 0.0 (*SD* = 0.2) in the quartile with the lowest GAD trait symptom severity to 3.0 (*SD* = 1.7) in the quartile with the highest. Correspondingly, the average GAD clinician severity rating increases from 0.1 (*SD* = 0.2) in the quartile with the lowest GAD trait symptom severity to 3.6 (*SD* = 1.6) in the quartile with the highest. Thus, those who score high on the GAD trait symptom severity factor (e.g., top quartile) tend to experience full criteria or greater GAD symptom severity the majority of time points (chronically at high levels of GAD symptom severity). For these participants, a time point with lower GAD symptom severity would be reflected in their occasion standing. Conversely, for those who score low on the GAD trait symptom severity factor (e.g., bottom quartile) the norm is to experience close to no GAD symptoms (chronically at low levels of GAD symptom severity). For these participants, a time point with higher GAD symptom severity would be reflected in their occasion standing. A similar pattern is observed for MDD (see Table 2).[Table-anchor tbl2]

When examining key study hypotheses using regression analyses (see Figure 2), models for each child outcome/maternal disorder pairing fit the data well: all CFI > .96, RMSEA < .049. However, when child outcomes were simultaneously regressed onto more than two occasion factors, or two occasion factors at adjacent time points, multicollinearity generally resulted in substantial increases in coefficient standard errors. Thus, for each child outcome/maternal disorder pairing, the child outcome at 24 months postpartum was simultaneously regressed onto the trait factor and pairs of nonadjacent occasion factors. Standardized regression coefficients for each child outcome/maternal disorder pairing are in Table 3 for the simultaneous regression of child outcomes on the trait factor as well as the 3- and 24-month postpartum occasion factors. Occasion factor pathways for 6, 10, and 14 months postpartum were not included because they were all nonsignificant, all *p* > .05, and comparable in magnitude to those reported for 3- and 24-month postpartum occasion factors.[Table-anchor tbl3]

As seen in Table 3, maternal report of child CBCL total problems, CBCL internalizing, CBCL externalizing, ECBQ frustration, and ECBQ soothability at 24 months postpartum demonstrated significant associations with maternal MDD and GAD trait factors, but were not significantly associated with occasion factors at 3, 6, 10, 14, or 24 months after accounting for the association with the trait factor. Maternal reports of child ECBQ attentional focusing and ECBQ attentional shifting at 24 months postpartum were not related to any facet of maternal MDD or GAD.

When considering the observational outcomes, neither occasion nor trait factors for maternal GAD were significantly related to child negative emotional tone or negative emotional reactivity at 24 months postpartum (see Table 3). However, the maternal MDD trait factor was significantly related to both child negative emotional tone and child negative emotional reactivity at 24 months postpartum, such that as the MDD trait factor increased, the child appeared more negative during the interaction tasks and was more negatively reactive to frustration. Maternal sensitivity was not significantly related to trait or occasion factors of maternal MDD or GAD.

The standardized pathway coefficients in Table 3 represent the change in standard deviations of the child outcome variable that are associated with a one standard deviation change in the predictor variable (when accounting for the relations between the other predictors and the child outcome). For example, when considering the prediction of child CBCL total problems from one’s standing on the portion of maternal MDD symptom severity that is stable from 3 to 24 months postpartum (trait factor), the standardized pathway coefficient, β_*T*_ is .38, *p* < .001. Thus, a one standard deviation increase in standing on the MDD symptom severity trait factor is associated with a .38 standard deviation increase in child CBCL total problems at 24 months postpartum when accounting for the relation between child total problems and standing on the MDD symptom severity occasion factors at 3 and 24 months postpartum.

Because maternal report of child CBCL total problems, CBCL internalizing, CBCL externalizing, ECBQ frustration, and ECBQ soothability at 24 months postpartum demonstrated significant associations with both maternal MDD and GAD trait factors, these child outcomes were next included in a combined model where they were simultaneously regressed onto both MDD and GAD trait factors. The model, which included both MDD and GAD TSO models (where MDD and GAD trait factors were allowed to correlate with one another, as were MDD and GAD occasion factors at each time point), was a borderline good fit to the data, CFI = .93, RMSEA = .069. After accounting for the relation between the GAD trait factor and the child outcome, the MDD trait factor independently predicted CBCL total problems, β_*T*_ = 0.44 (*SE* = 0.18), *p* = .016, ECBQ frustration, β_*T*_ = 0.49 (*SE* = 0.21), *p* = .020, and nearly was a significant predictor of ECBQ soothability, β_*T*_ = −0.35 (*SE* = 0.19), *p* = .062. However, after accounting for the relation between the MDD trait factor and the child outcome, the GAD trait factor was not a significant predictor of any of the maternal report outcomes, all β_*T*_ < |.08|, all *p* > .05. Because none of the observational outcomes were significantly predicted by both MDD and GAD TSO components, the combined model was not used to examine these outcomes.

## Discussion

The current study found that persistent maternal anxiety and depressive symptom severity during the first two years postpartum was related to maternal reports of poorer behavior and emotional negativity in their 24-month-old children. However, when persistent maternal anxiety and depressive symptom severity were both included as predictors, persistent anxiety did not independently predict any of the maternal reported child outcomes above and beyond persistent maternal depression, whereas persistent maternal depression independently predicted total child problems and frustration (and nearly soothability) after accounting for persistent maternal anxiety. Further, persistent maternal depression, but not anxiety, was related to observational evidence for poorer child negative emotional tone and negative emotional reactivity during a stressful task. These data highlight the potential negative impact of persistent maternal depression and anxiety on children’s emotional and behavioral outcomes, with more robust evidence for the impact of maternal depression than anxiety. The findings are noteworthy given prior evidence that higher levels of negative emotional reactivity as early as 14 or 21 months is related to poorer emotional development later in childhood (e.g., Kagan et al., 1998). These findings indicate the importance of intervening early and, perhaps, repeatedly for those mothers at risk for persistent depression and anxiety.

Despite the fact that maternal depression was less stable than maternal anxiety, the stable component of maternal depression yielded more robust effects upon children’s behavior and emotional negativity than did anxiety, in that the effects extended beyond maternal self-report to independent laboratory observations. Additionally, only persistent maternal depression was independently related to maternal-reported child outcomes when both persistent maternal depression and anxiety were simultaneously examined as predictors. Anxiety and depression are both characterized by negative interpretation biases of ambiguous information (Mathews & MacLeod, 2005). Children’s emotions and behaviors can be difficult to interpret and previous studies have shown that reports of children’s emotions and regulatory skills from depressed and anxious mothers may be negatively biased (Müller, Achtergarde, & Furniss, 2011). However, the current findings suggest that the effects of persistent maternal depression were not limited to maternal biases in reporting since they were confirmed by laboratory observations.

In contrast, maternal anxiety was not related to independent observations of child outcomes. Maternal report of child emotions and emotional negativity is important in its own right for several reasons. First, maternal report represents an estimate of the child based on experiences covering more situations, and likely more stressful situations, than are induced in laboratory paradigms. Therefore, maternal report may represent a more generalizable view of the child than does laboratory observation. It is possible that the laboratory tasks were insufficiently sensitive to measure child outcomes. Indeed, had the laboratory tasks been more frightening than frustrating the effects of persistent maternal anxiety may have become evident. Second, even if maternal report is biased, the mother’s view of her child’s emotional state influences parenting styles in ways that contribute to child difficulties. For example, mothers of anxious children expect their children to interpret ambiguous situations as threatening and to avoid them, and they actively encourage avoidance (Dadds, Barrett, Rapee, & Ryan, 1996; Murray et al., 2014) which contributes to further anxiety. In this way persistent maternal anxiety, through its impact on mothers’ perception of their child’s behavior and emotional negativity, could have a negative impact on parenting styles and the child’s long term development.

High levels of negative affect characterize both anxiety and depression, but deficits in positive affect are more uniquely tied to depression (Watson, 2005). Low positive affect is associated with symptoms of loss of interest, drive, and pleasure. High negative affect is associated with symptoms of anxiety, sadness, anger, and guilt. The current findings may suggest that the parenting effects of low positive affect may be more detrimental to the child’s observable emotional negativity than the parenting effects of high negative affect, or that the combination of high negative affect and low positive affect is more detrimental than high negative affect alone. As noted, there is extensive evidence that maternal depression, especially persistent depression, has a negative impact on child development (Murray et al., 2010) and parental emotional scaffolding (Tronick & Weinberg, 1997), which in turn predicts child emotional dysregulation (Murray, Woolgar, Cooper, & Hipwell, 2001). In contrast, results from maternal anxiety are more limited and mixed.

Although the current findings may suggest parenting effects of low positive affect are more detrimental to the child’s observable emotional negativity than the parenting effects of high negative affect, another possibility is that the comorbidity of depression and anxiety plays a role in the relation between maternal psychopathology and child outcomes. Psychopathological comorbidity is associated with poorer functioning in adults (e.g., Kessler et al., 1996; Mineka et al., 1998), comorbid anxiety and depression are more chronic than either syndrome alone (Merikangas et al., 2003), and at least one study found that the relation between maternal depression and child functioning was specific to mothers with comorbid diagnoses (Carter et al., 2001). In the present sample, symptoms of maternal depression were significantly less stable than those of maternal anxiety. Thus, those with high levels on the stable depressive symptom factor would be more likely to have high levels on the stable anxiety symptom factor than would those with high levels on the stable anxiety symptom factor be likely to have high levels on the stable depressive symptom factor. In accord, as seen in Table 2, those in the highest quartile on the MDD trait factor were diagnosed with GAD an average of 2.39 (*SD* = 1.91) of the 5 time points, whereas those in the highest quartile on the GAD trait factor were diagnosed with MDD an average of 1.88 (*SD* = 1.59) of the 5 time points. Consequently, the finding that only the depressive symptom severity trait factor predicted observable child outcomes and independently predicted maternal reports of emotional negativity and behavioral outcomes are most likely due to the depression trait factor capturing increased chronicity of symptom co-occurrence to a greater extent than the anxiety trait factor.

Notably, chronicity of maternal anxiety and depression did not predict maternal sensitivity at 24 months. One possible explanation is that the mothers and children were observed in a relatively unchallenging environment. Evidence indicates that more negative parental behaviors only emerge under stressful conditions (Stein et al., 2012), and replication under such conditions may yield significant findings.

Additionally, there was little evidence that timing of maternal depressive or anxiety symptoms moderated the relation between maternal psychopathology and child outcomes for the period under consideration. Specifically, the occasion factors at 3, 6, 10, 14, and 24 months did not predict child outcomes for either depression or anxiety after accounting for the trait factors. Thus, unlike Brennan and colleagues (2000), we did not find that elevations in depressive symptoms specific to 6 months postpartum were associated with poorer child outcomes. Although Brennan and colleagues primary outcome was one used in the present study, the total behavior problem score from the CBCL, they operationalized maternal depression differently and examined child behavior at an older age. Further evaluation is needed to determine if there are critical time periods when maternal pathology may have a greater influence on child development.

Strengths of our study include the evaluation of maternal anxiety in addition to the more commonly studied maternal depression. Through the application of TSO modeling, we evaluated the effects of the stable, trait component of maternal anxiety and depression (uncontaminated by occasion-specific variance at the point of assessment) on child outcomes over the first two years of life. This approach allowed us to evaluate the role of maternal psychopathology chronicity in a manner that better reflects how relative standing on psychopathological constructs is expressed with time (Prenoveau et al., 2013).

TSO modeling also enabled investigation of the relation between child outcomes and maternal occasion-specific depression and anxiety after accounting for the relation between persistent maternal depression and anxiety and child outcomes. With only one exception, there was no relation between the 24-month child outcomes and occasion-specific maternal pathology (when child outcomes were assessed) after accounting for persistent psychopathological symptom severity. This highlights the importance of persistent relative to intermittent maternal symptom severity.

Limitations include all observational assessments being carried out in a laboratory which, although providing assessment standardization, may limit variability of the kind seen in real life settings. Child outcomes were only assessed at one time point and different outcomes may be observed over development. We did not find specific difficulties in maternal behavior that explained children’s behavioral problems or emotional negativity. This could be a limitation of our assessment design, in that observations of maternal behavior were made in a single laboratory session under relatively low stress conditions. Conceivably, naturalistic home observations would have provided a better account of the causal relationships. GAD and MDD rates in the present subsample (*n* = 296) were greater than that of the general maternal population, which may impact the magnitudes of the observed relations. Additionally, the sample consisted primarily of Caucasian mothers. Future work should extend these findings by examining them in a more representative sample of postpartum mothers. Another potential limitation is that inadequate sample sizes for structural equation modeling can result in biased parameter estimates and standard errors. Although the present study meets suggested ‘rules-of-thumb’ such as a minimum sample size of 100 or 200 (Boomsma, 1985) and between 5 to 10 observations per estimated parameter (e.g., Bentler & Chou, 1987), recent work demonstrates that such rules-of-thumb are problematic as sample size requirements depend on a number of model features (e.g., number of indicators and factors, magnitude of factor loadings and path coefficients; Wolf, Harrington, Clark, & Miller, 2013). Given the complexity of the models tested, future work could employ larger sample sizes to reduce the likelihood of introducing bias.

In conclusion, the current findings highlight the importance of early and if necessary sustained intervention for mothers who are at risk for persistent depression, and possibly also for those at risk for persistent anxiety, in the early years of their children’s lives. Findings are in line with recent work indicating that brief episodes of postnatal depression or anxiety may not have negative effects on child development (Goodman et al., 2011). Although it is, of course, important to offer help to any parent who experiences significant postnatal depression or anxiety, efforts to prevent adverse effects on children should likely be concentrated on parents with persistent psychopathology.

## Supplementary Material

10.1037/dev0000221.supp

## Figures and Tables

**Table 1 tbl1:** Demographics by Diagnostic Status at Three Months Postpartum

Demographic variable and variable level	Co-occurring GAD and MDD (*n* = 41)	GAD only (*n* = 80)	MDD only (*n* = 40)	No disorder (*n* = 135)	Statistics
Mother age in years, *M* (*SD*)	32.5 (5.3)	31.8 (5.1)	32.3 (5.6)	32.6 (4.8)	*F*(3, 292) = 0.4, *p* = .74
Infant age in months, *M* (*SD*)	3.5 (.9)	3.7 (0.8)	3.7 (1.2)	3.5 (0.6)	*F*(3, 319) = 1.3, *p* = .26
Infant sex, frequency (%)					
Female	17 (41.5%)	40 (50%)	21 (52.5%)	70 (51.9%)	
Male	24 (58.5%)	40 (50%)	19 (47.5%)	65 (48.1%)	χ^2^(3) = 1.5, *p* = .69
Infant birth order, frequency (%)				
First born	19 (46.3%)	48 (60.0%)	19 (47.5%)	94 (69.6%)	
Not first born	22 (53.7%)	32 (40.0%)	21 (52.5%)	41 (30.4%)	χ^2^(3) = 11.0, *p* = .01
Mother marital status, frequency (%)					
Married	19 (46.3%)	48 (60.0%)	24 (60.0%)	93 (68.9%)	
Other	5 (12.2%)	22 (27.5%)	9 (22.5%)	25 (18.5%)	
Missing	17 (41.5%)	10 (12.5%)	7 (17.5%)	17 (12.6%)	χ^2^(3) = 23.0, *p* = .001
*Note.* GAD = generalized anxiety disorder; MDD = major depressive disorder.					

**Table 2 tbl2:** Number of Time Points With, and Clinician Severity Ratings for, Maternal MDD and GAD by Relative Standing on MDD and GAD TSO Model Trait Factor

Maternal relative standing on symptom severity trait factor	Number of time points with MDD, *M* (*SD*)	Clinician severity rating across time points for MDD, *M* (*SD*)	Number of time points with GAD, *M* (*SD*)	Clinician severity rating across time points for GAD, *M* (*SD*)
MDD trait factor standing				
Lowest quartile (*n* = 74)	0.05 (0.28)	0.11 (0.39)	0.24 (0.76)	0.33 (0.94)
Mid–low quartile (*n* = 74)	0.11 (0.35)	0.23 (0.51)	0.35 (0.75)	0.51 (1.04)
Mid–high quartile (*n* = 74)	0.68 (0.91)	1.40 (1.26)	1.80 (1.53)	2.56 (1.57)
Highest quartile (*n* = 74)	2.42 (1.38)	3.25 (1.45)	2.39 (1.91)	2.80 (2.09)
GAD trait factor standing				
Lowest quartile (*n* = 74)	0.03 (0.23)	0.06 (0.27)	0.04 (0.20)	0.06 (0.25)
Mid–low quartile (*n* = 74)	0.22 (0.67)	0.43 (0.83)	0.36 (0.71)	0.57 (0.94)
Mid–high quartile (*n* = 74)	1.14 (1.19)	1.87 (1.45)	1.35 (1.33)	2.09 (1.70)
Highest quartile (*n* = 74)	1.88 (1.59)	2.76 (1.72)	3.03 (1.66)	3.55 (1.61)
*Note*. TSO = Trait–state–occasion; MDD = major depressive disorder; GAD = generalized anxiety disorder.				

**Table 3 tbl3:** Standardized Coefficients for Pathways Between Child Outcomes at 24 Months Postpartum and TSO Factors for Maternal MDD and GAD

Child outcome at 24 months postpartum	Standardized pathway coefficient (*SE*)					
	Maternal MDD			Maternal GAD		
	β_*O*_3 *M*−*P*__	β_*O*_24 *M*−*P*__	β_*T*_	β_*O*_3 *M*−*P*__	β_*O*_24 *M*−*P*__	β_*T*_
Maternal report						
CBCL total problems	−.02 (.10)	.08 (.13)	.38*** (.09)	.08 (.08)	−.08 (.08)	.32*** (.06)
CBCL internalizing	−.01 (.10)	.09 (.14)	.30** (.10)	.06 (.08)	−.02 (.08)	.27*** (.07)
CBCL externalizing	−.02 (.10)	.12 (.13)	.31** (.10)	.08 (.08)	−.05 (.08)	.29*** (.07)
CBCL attention	−.02 (.10)	.20 (.14)	.17 (.11)	.09 (.08)	.06 (.09)	.20** (.07)
ECBQ frustration	.14 (.11)	.03 (.15)	.41*** (.11)	.06 (.08)	−.02 (.09)	.33*** (.07)
ECBQ soothability	−.12 (.11)	.10 (.14)	−.30** (.10)	−.10 (.08)	.01 (.08)	−.23*** (.07)
ECBQ attentional focusing	.04 (.11)	−.03 (.15)	.00 (.12)	−.06 (.09)	.12 (.08)	−.01 (.08)
ECBQ attentional shifting	−.05 (.11)	−.01 (.16)	−.13 (.13)	−.18 (.10)	.02 (.09)	−.08 (.08)
Observational						
Child ET	.14 (.11)	.24 (.15)	−.28* (.12)	.03 (.08)	.03 (.08)	−.12 (.07)
Child ER	.22* (.10)	.22 (.13)	−.21* (.10)	.01 (.09)	.03 (.09)	.01 (.08)
Maternal sensitivity	.04 (.11)	.00 (.14)	−.08 (.11)	−.09 (.08)	−.13 (.08)	−.03 (.07)
*Note.* Because marital status and infant birth order are potentially confounded with MDD and GAD symptom severity, they were included as predictors of child outcomes in all models. CBCL = Child Behavior Checklist for Ages 1.5–5; ECBQ = Early Childhood Behavior Questionnaire; ER = negative emotional reactivity; ET = negative emotional tone; MDD = major depressive disorder; GAD = generalized anxiety disorder; β = standardized coefficient for the pathway from the trait–state–occasion (TSO) model component denoted in the β subscript to the child outcome under consideration (see Figure 1). β subscripts: O_3M-P_ = TSO occasion factor at 3 months postpartum; O_24M-P_ = TSO occasion factor at 24 months postpartum; T = TSO trait factor.						
* *p* < .05. ** *p* < .01. *** *p* < .001.
