# Supplementary material for: Maternal Postnatal Depression and Anxiety and Their Association With Child Emotional Negativity and Behavior Problems at Two Years
Source: Dev Psychol. 2017 Jan;53(1):50–62. doi: 10.1037/dev0000221 (PMC5191902; doi:10.1037/dev0000221)
Supplement: Supplementary file 1 [file z2p011163782so1.doc]

**Supplemental Materials**

**Maternal Postnatal Depression and Anxiety and Their Association With Child Emotional Negativity and Behavior Problems at Two Years**

**by J. M. Prenoveau et al., 2016, *Developmental Psychology***

**http://dx.doi.org/10.1037/dev0000221**

*Means and Standard Deviations for, and Correlations among, Maternal MDD and GAD Symptom Severity Measures at 3, 6, 10, 14, and 24 Months Postpartum and Child Outcomes at 24 Months Postpartum*

|  | MDD CSR 3MP | EPDS1 3MP | EPDS2 3MP | MDD CSR 6MP | EPDS1 6MP | EPDS2 6MP | MDD CSR 10MP | EPDS1 10MP | EPDS2 10MP | MDD CSR 14MP | EPDS1 14MP | EPDS2 14MP | MDD CSR 24MP | EPDS1 24MP | EPDS2 24MP | GAD CSR 3MP | GADQ1 3MP | GADQ2 3MP | GAD CSR 6MP | GADQ1 6MP | GADQ2 6MP | GAD CSR 10MP | GADQ1 10MP | GADQ2 10MP | GAD CSR 14MP | GADQ1 14MP | GADQ2 14MP | GAD CSR 24MP | GADQ1 24MP | GADQ2 24MP | Total Problems | Internalizing | Externalizing | Attention | Frustration | Soothability | Attentional Focusing | Attentional Shifting | Child Negative Emotional Tone | Child Negative Emotional Reactivity | Maternal Sensitivity |
| --- | --- | --- | --- | --- | --- | --- | --- | --- | --- | --- | --- | --- | --- | --- | --- | --- | --- | --- | --- | --- | --- | --- | --- | --- | --- | --- | --- | --- | --- | --- | --- | --- | --- | --- | --- | --- | --- | --- | --- | --- | --- |
| MDD CSR 3MP |  | .43^**^ | .44^**^ | .66^**^ | .49^**^ | .54^**^ | .49^**^ | .43^**^ | .44^**^ | .58^**^ | .44^**^ | .51^**^ | .44^**^ | .31^**^ | .35^**^ | .17^**^ | .27^**^ | .35^**^ | .26^**^ | .49^**^ | .56^**^ | .19^**^ | .48^**^ | .48^**^ | .28^**^ | .50^**^ | .50^**^ | .26^**^ | .35^**^ | .38^**^ | .13 | .08 | .14^*^ | .06 | .23^**^ | -.17^*^ | .05 | -.06 | -.07 | .05 | -.03 |
| EPDS1 3MP |  |  | .79^**^ | .26^**^ | .42^**^ | .39^**^ | .31^**^ | .39^**^ | .35^**^ | .25^**^ | .37^**^ | .37^**^ | .14^*^ | .26^**^ | .27^**^ | .17^**^ | .63^**^ | .67^**^ | .15^*^ | .35^**^ | .41^**^ | .14^*^ | .32^**^ | .40^**^ | .12 | .29^**^ | .41^**^ | .16^*^ | .21^**^ | .33^**^ | .26^**^ | .22^**^ | .17^*^ | .10 | .27^**^ | -.30^**^ | -.02 | -.11 | -.05 | -.11 | -.04 |
| EPDS2 3MP |  |  |  | .29^**^ | .34^**^ | .39^**^ | .31^**^ | .30^**^ | .35^**^ | .23^**^ | .27^**^ | .28^**^ | .12 | .18^**^ | .18^*^ | .15^**^ | .62^**^ | .70^**^ | .12^*^ | .34^**^ | .39^**^ | .09 | .25^**^ | .37^**^ | .12 | .27^**^ | .36^**^ | .16^*^ | .19^**^ | .29^**^ | .25^**^ | .20^**^ | .18^**^ | .14^*^ | .24^**^ | -.20^**^ | .01 | -.11 | -.04 | -.07 | -.05 |
| MDD CSR 6MP |  |  |  |  | .63^**^ | .69^**^ | .67^**^ | .50^**^ | .55^**^ | .65^**^ | .56^**^ | .60^**^ | .61^**^ | .42^**^ | .48^**^ | .24^**^ | .17^**^ | .19^**^ | .43^**^ | .61^**^ | .64^**^ | .31^**^ | .50^**^ | .58^**^ | .41^**^ | .60^**^ | .57^**^ | .39^**^ | .44^**^ | .50^**^ | .20^**^ | .15^*^ | .18^**^ | .10 | .23^**^ | -.18^*^ | .03 | .00 | -.14^*^ | -.06 | -.05 |
| EPDS1 6MP |  |  |  |  |  | .75^**^ | .58^**^ | .72^**^ | .60^**^ | .50^**^ | .61^**^ | .52^**^ | .45^**^ | .49^**^ | .44^**^ | .28^**^ | .30^**^ | .30^**^ | .36^**^ | .63^**^ | .71^**^ | .39^**^ | .61^**^ | .67^**^ | .35^**^ | .50^**^ | .59^**^ | .41^**^ | .46^**^ | .53^**^ | .33^**^ | .27^**^ | .25^**^ | .17^*^ | .41^**^ | -.26^**^ | -.06 | -.14 | -.16^*^ | -.07 | -.07 |
| EPDS2 6MP |  |  |  |  |  |  | .62^**^ | .65^**^ | .73^**^ | .58^**^ | .56^**^ | .61^**^ | .55^**^ | .52^**^ | .63^**^ | .28^**^ | .28^**^ | .31^**^ | .38^**^ | .64^**^ | .71^**^ | .34^**^ | .61^**^ | .68^**^ | .32^**^ | .56^**^ | .59^**^ | .43^**^ | .53^**^ | .57^**^ | .21^**^ | .13 | .19^**^ | .08 | .37^**^ | -.22^**^ | .00 | -.12 | -.11 | -.03 | -.01 |
| MDD CSR 10MP |  |  |  |  |  |  |  | .65^**^ | .73^**^ | .66^**^ | .55^**^ | .56^**^ | .60^**^ | .45^**^ | .42^**^ | .28^**^ | .23^**^ | .21^**^ | .43^**^ | .59^**^ | .63^**^ | .44^**^ | .71^**^ | .66^**^ | .41^**^ | .62^**^ | .59^**^ | .48^**^ | .52^**^ | .51^**^ | .23^**^ | .13^*^ | .23^**^ | .13 | .21^**^ | -.20^**^ | .02 | .03 | .00 | -.10 | -.05 |
| EPDS1 10MP |  |  |  |  |  |  |  |  | .74^**^ | .59^**^ | .66^**^ | .60^**^ | .44^**^ | .43^**^ | .39^**^ | .28^**^ | .29^**^ | .30^**^ | .36^**^ | .51^**^ | .65^**^ | .40^**^ | .68^**^ | .69^**^ | .37^**^ | .51^**^ | .60^**^ | .43^**^ | .43^**^ | .48^**^ | .28^**^ | .18^*^ | .21^**^ | .14 | .25^**^ | -.19^**^ | .02 | -.03 | -.03 | -.15^*^ | -.05 |
| EPDS2 10MP |  |  |  |  |  |  |  |  |  | .59^**^ | .50^**^ | .62^**^ | .46^**^ | .36^**^ | .46^**^ | .32^**^ | .27^**^ | .29^**^ | .35^**^ | .53^**^ | .64^**^ | .39^**^ | .67^**^ | .70^**^ | .33^**^ | .52^**^ | .55^**^ | .42^**^ | .39^**^ | .47^**^ | .30^**^ | .16^*^ | .28^**^ | .15^*^ | .28^**^ | -.21^**^ | .01 | -.12 | .02 | -.11 | -.02 |
| MDD CSR 14MP |  |  |  |  |  |  |  |  |  |  | .72^**^ | .76^**^ | .62^**^ | .46^**^ | .46^**^ | .36^**^ | .32^**^ | .28^**^ | .44^**^ | .57^**^ | .60^**^ | .47^**^ | .67^**^ | .63^**^ | .54^**^ | .78^**^ | .70^**^ | .48^**^ | .54^**^ | .55^**^ | .27^**^ | .16^*^ | .29^**^ | .17^*^ | .20^*^ | -.15^*^ | .00 | -.11 | -.13 | -.15^*^ | -.08 |
| EPDS1 14MP |  |  |  |  |  |  |  |  |  |  |  | .72^**^ | .47^**^ | .54^**^ | .41^**^ | .33^**^ | .27^**^ | .32^**^ | .41^**^ | .52^**^ | .63^**^ | .51^**^ | .64^**^ | .68^**^ | .53^**^ | .71^**^ | .75^**^ | .42^**^ | .46^**^ | .57^**^ | .37^**^ | .29^**^ | .28^**^ | .22^**^ | .31^**^ | -.21^**^ | -.09 | -.03 | -.17^*^ | -.17^*^ | -.03 |
| EPDS2 14MP |  |  |  |  |  |  |  |  |  |  |  |  | .51^**^ | .52^**^ | .55^**^ | .37^**^ | .38^**^ | .32^**^ | .43^**^ | .52^**^ | .62^**^ | .42^**^ | .58^**^ | .67^**^ | .48^**^ | .70^**^ | .72^**^ | .48^**^ | .50^**^ | .61^**^ | .26^**^ | .15 | .22^**^ | .14 | .24^**^ | -.14 | .03 | -.07 | -.06 | -.15 | -.05 |
| MDD CSR 24MP |  |  |  |  |  |  |  |  |  |  |  |  |  | .64^**^ | .68^**^ | .37^**^ | .20^**^ | .20^**^ | .49^**^ | .55^**^ | .55^**^ | .40^**^ | .50^**^ | .54^**^ | .41^**^ | .61^**^ | .55^**^ | .59^**^ | .55^**^ | .63^**^ | .29^**^ | .20^**^ | .31^**^ | .23^**^ | .34^**^ | -.09 | -.06 | -.08 | -.13 | -.09 | -.08 |
| EPDS1 24MP |  |  |  |  |  |  |  |  |  |  |  |  |  |  | .73^**^ | .38^**^ | .22^**^ | .21^**^ | .43^**^ | .45^**^ | .51^**^ | .43^**^ | .43^**^ | .48^**^ | .46^**^ | .54^**^ | .52^**^ | .53^**^ | .58^**^ | .71^**^ | .34^**^ | .29^**^ | .29^**^ | .22^**^ | .24^**^ | -.21^**^ | -.04 | -.06 | -.01 | -.02 | -.05 |
| EPDS2 24MP |  |  |  |  |  |  |  |  |  |  |  |  |  |  |  | .38^**^ | .26^**^ | .25^**^ | .39^**^ | .50^**^ | .56^**^ | .39^**^ | .42^**^ | .53^**^ | .40^**^ | .54^**^ | .50^**^ | .55^**^ | .59^**^ | .73^**^ | .26^**^ | .20^**^ | .25^**^ | .19^**^ | .27^**^ | -.19^**^ | .01 | -.03 | -.07 | .00 | -.07 |
| GAD CSR 3MP |  |  |  |  |  |  |  |  |  |  |  |  |  |  |  |  | .41^**^ | .31^**^ | .82^**^ | .57^**^ | .56^**^ | .72^**^ | .51^**^ | .53^**^ | .67^**^ | .48^**^ | .56^**^ | .68^**^ | .52^**^ | .54^**^ | .30^**^ | .22^**^ | .29^**^ | .23^**^ | .28^**^ | -.15^*^ | -.10 | -.10 | -.05 | -.01 | -.09 |
| GADQ1 3MP |  |  |  |  |  |  |  |  |  |  |  |  |  |  |  |  |  | .81^**^ | .27^**^ | .43^**^ | .45^**^ | .23^**^ | .38^**^ | .44^**^ | .27^**^ | .41^**^ | .47^**^ | .29^**^ | .34^**^ | .39^**^ | .20^**^ | .13 | .19^**^ | .18^**^ | .08 | -.10 | -.08 | -.15 | -.02 | -.05 | -.13 |
| GADQ2 3MP |  |  |  |  |  |  |  |  |  |  |  |  |  |  |  |  |  |  | .23^**^ | .42^**^ | .45^**^ | .21^**^ | .33^**^ | .44^**^ | .23^**^ | .33^**^ | .44^**^ | .27^**^ | .28^**^ | .34^**^ | .19^**^ | .11 | .19^**^ | .11 | .27^**^ | -.19^**^ | -.03 | -.15^*^ | -.12 | -.13 | -.14^*^ |
| GAD CSR 6MP |  |  |  |  |  |  |  |  |  |  |  |  |  |  |  |  |  |  |  | .62^**^ | .62^**^ | .80^**^ | .61^**^ | .60^**^ | .78^**^ | .56^**^ | .58^**^ | .76^**^ | .58^**^ | .57^**^ | .28^**^ | .22^**^ | .28^**^ | .22^**^ | .25^**^ | -.11 | -.10 | -.05 | -.10 | -.03 | -.09 |
| GADQ1 6MP |  |  |  |  |  |  |  |  |  |  |  |  |  |  |  |  |  |  |  |  | .83^**^ | .64^**^ | .75^**^ | .75^**^ | .59^**^ | .71^**^ | .69^**^ | .65^**^ | .70^**^ | .68^**^ | .22^**^ | .16^*^ | .22^**^ | .11 | .32^**^ | -.17^*^ | .05 | -.06 | -.12 | .04 | -.05 |
| GADQ2 6MP |  |  |  |  |  |  |  |  |  |  |  |  |  |  |  |  |  |  |  |  |  | .60^**^ | .77^**^ | .90^**^ | .61^**^ | .67^**^ | .82^**^ | .62^**^ | .65^**^ | .74^**^ | .36^**^ | .28^**^ | .34^**^ | .21^**^ | .43^**^ | -.24^**^ | -.05 | -.16^*^ | -.06 | -.01 | -.05 |
| GAD CSR 10MP |  |  |  |  |  |  |  |  |  |  |  |  |  |  |  |  |  |  |  |  |  |  | .67^**^ | .65^**^ | .83^**^ | .58^**^ | .60^**^ | .74^**^ | .59^**^ | .61^**^ | .32^**^ | .25^**^ | .32^**^ | .24^**^ | .30^**^ | -.11 | -.08 | -.08 | -.05 | .05 | -.03 |
| GADQ1 10MP |  |  |  |  |  |  |  |  |  |  |  |  |  |  |  |  |  |  |  |  |  |  |  | .83^**^ | .62^**^ | .71^**^ | .70^**^ | .62^**^ | .65^**^ | .61^**^ | .30^**^ | .22^**^ | .28^**^ | .19^*^ | .34^**^ | -.21^**^ | -.05 | -.13 | -.01 | .02 | -.01 |
| GADQ2 10MP |  |  |  |  |  |  |  |  |  |  |  |  |  |  |  |  |  |  |  |  |  |  |  |  | .63^**^ | .70^**^ | .85^**^ | .62^**^ | .60^**^ | .74^**^ | .41^**^ | .30^**^ | .35^**^ | .25^**^ | .39^**^ | -.27^**^ | -.02 | -.10 | .00 | .01 | .02 |
| GAD CSR 14MP |  |  |  |  |  |  |  |  |  |  |  |  |  |  |  |  |  |  |  |  |  |  |  |  |  | .67^**^ | .66^**^ | .77^**^ | .66^**^ | .63^**^ | .30^**^ | .23^**^ | .33^**^ | .23^**^ | .24^**^ | -.10 | -.02 | -.10 | -.14^*^ | -.02 | -.05 |
| GADQ1 14MP |  |  |  |  |  |  |  |  |  |  |  |  |  |  |  |  |  |  |  |  |  |  |  |  |  |  | .81^**^ | .65^**^ | .73^**^ | .74^**^ | .25^**^ | .16 | .25^**^ | .23^**^ | .29^**^ | -.17^*^ | .01 | .00 | -.10 | -.09 | .02 |
| GADQ2 14MP |  |  |  |  |  |  |  |  |  |  |  |  |  |  |  |  |  |  |  |  |  |  |  |  |  |  |  | .61^**^ | .65^**^ | .77^**^ | .36^**^ | .27^**^ | .33^**^ | .27^**^ | .35^**^ | -.21^**^ | .03 | -.01 | -.11 | -.11 | -.03 |
| GAD CSR 24MP |  |  |  |  |  |  |  |  |  |  |  |  |  |  |  |  |  |  |  |  |  |  |  |  |  |  |  |  | .80^**^ | .72^**^ | .23^**^ | .16^*^ | .24^**^ | .21^**^ | .29^**^ | -.15^*^ | .03 | -.04 | -.07 | -.01 | -.09 |
| GADQ1 24MP |  |  |  |  |  |  |  |  |  |  |  |  |  |  |  |  |  |  |  |  |  |  |  |  |  |  |  |  |  | .79^**^ | .19^*^ | .17^*^ | .22^**^ | .21^**^ | .25^**^ | -.15^*^ | -.03 | -.09 | -.05 | .05 | -.11 |
| GADQ2 24MP |  |  |  |  |  |  |  |  |  |  |  |  |  |  |  |  |  |  |  |  |  |  |  |  |  |  |  |  |  |  | .38^**^ | .32^**^ | .33^**^ | .28^**^ | .34^**^ | -.24^**^ | -.07 | -.10 | -.02 | .02 | -.08 |
| Total Problems |  |  |  |  |  |  |  |  |  |  |  |  |  |  |  |  |  |  |  |  |  |  |  |  |  |  |  |  |  |  |  | .84^**^ | .88^**^ | .67^**^ | .58^**^ | -.47^**^ | -.35^**^ | -.38^**^ | -.06 | -.20^**^ | -.06 |
| Internalizing |  |  |  |  |  |  |  |  |  |  |  |  |  |  |  |  |  |  |  |  |  |  |  |  |  |  |  |  |  |  |  |  | .58^**^ | .43^**^ | .44^**^ | -.43^**^ | -.22^**^ | -.27^**^ | -.03 | -.17^*^ | -.04 |
| Externalizing |  |  |  |  |  |  |  |  |  |  |  |  |  |  |  |  |  |  |  |  |  |  |  |  |  |  |  |  |  |  |  |  |  | .76^**^ | .59^**^ | -.38^**^ | -.41^**^ | -.40^**^ | -.10 | -.18^*^ | -.06 |
| Attention |  |  |  |  |  |  |  |  |  |  |  |  |  |  |  |  |  |  |  |  |  |  |  |  |  |  |  |  |  |  |  |  |  |  | .36^**^ | -.29^**^ | -.52^**^ | -.34^**^ | -.11 | -.20^**^ | -.07 |
| Frustration |  |  |  |  |  |  |  |  |  |  |  |  |  |  |  |  |  |  |  |  |  |  |  |  |  |  |  |  |  |  |  |  |  |  |  | -.52^**^ | -.36^**^ | -.41^**^ | -.12 | -.20^*^ | .00 |
| Soothability |  |  |  |  |  |  |  |  |  |  |  |  |  |  |  |  |  |  |  |  |  |  |  |  |  |  |  |  |  |  |  |  |  |  |  |  | .17^*^ | .38^**^ | .16^*^ | .20^**^ | .06 |
| Attentional Focusing |  |  |  |  |  |  |  |  |  |  |  |  |  |  |  |  |  |  |  |  |  |  |  |  |  |  |  |  |  |  |  |  |  |  |  |  |  | .30^**^ | .04 | .08 | .09 |
| Attentional Shifting |  |  |  |  |  |  |  |  |  |  |  |  |  |  |  |  |  |  |  |  |  |  |  |  |  |  |  |  |  |  |  |  |  |  |  |  |  |  | .19^*^ | .11 | .12 |
| Child Negative Emotional Tone |  |  |  |  |  |  |  |  |  |  |  |  |  |  |  |  |  |  |  |  |  |  |  |  |  |  |  |  |  |  |  |  |  |  |  |  |  |  |  | .62^**^ | .12 |
| Child Negative Emotional Reactivity |  |  |  |  |  |  |  |  |  |  |  |  |  |  |  |  |  |  |  |  |  |  |  |  |  |  |  |  |  |  |  |  |  |  |  |  |  |  |  |  | -.11 |
| Maternal Sensitivity |  |  |  |  |  |  |  |  |  |  |  |  |  |  |  |  |  |  |  |  |  |  |  |  |  |  |  |  |  |  |  |  |  |  |  |  |  |  |  |  |  |
| Mean | 1.5 | 1.7 | 2.0 | 1.1 | 1.6 | 1.8 | 1.2 | 1.4 | 1.6 | 1.2 | 1.5 | 1.6 | 1.2 | 1.4 | 1.6 | 1.9 | 1.7 | 5.5 | 1.5 | 1.7 | 5.1 | 1.4 | 1.5 | 4.6 | 1.2 | 1.4 | 4.6 | 1.6 | 1.4 | 4.4 | 34.3 | 7.8 | 13.7 | 2.8 | 3.3 | 5.6 | 4.4 | 4.6 | 3.8 | 4.3 | 4.0 |
| Standard Deviation | 2.3 | 2.0 | 2.0 | 1.8 | 1.9 | 1.8 | 1.9 | 1.8 | 1.7 | 1.9 | 1.8 | 1.6 | 1.8 | 1.7 | 1.5 | 2.3 | 1.9 | 3.6 | 2.0 | 1.9 | 3.4 | 2.0 | 1.9 | 3.4 | 1.9 | 1.9 | 3.6 | 2.1 | 1.9 | 3.4 | 16.8 | 5.4 | 7.1 | 1.8 | 0.9 | 0.6 | 0.8 | 0.6 | 0.7 | 0.9 | 0.6 |

*Note.* MDD = Major Depressive Disorder; CSR = clinician severity rating; MP = months postpartum; EPDS = Edinburgh Postnatal Depression Scale; GAD = Generalized Anxiety Disorder; GADQ = Generalized Anxiety Disorder Questionnaire.

^*^*p* < .05. ^**^*p* < .01. ^***^*p* < .001.
